# A Clinical Lecture on Pneumonia

**Published:** 1888-05

**Authors:** John A. Robison

**Affiliations:** Chicago, Ill.; Professor of Materia Medica and Therapeutics in Woman’s Medical College, Attending Physician to the County Hospital, etc.


### Clinic Reports.

### CLINICAL LECTURE ON PNEUMONIA.

BY JOHN A. ROBISON, A. M., M. D.,

Professor of Materia Medica and Therapeutics in Woman's Medical College, Attending Physician to the County Hospital, etc.

DELIVERED AT THE COOK COUNTY HOSPITAL.

Reported for The Atlanta Medical and Surgical Journal by William Whitford, M. D.

Gentlemen—I show you to-day a group of cases of pneumonia in which convalescence is established.

### DOUBLE PNEUMONIA.

Case 1. *History on Admission.*—Patient sick four days before admission into the hospital; works in a foundry; German by birth; age 23. Family history negative. Personal history: Has had chills and fever for a long time; otherwise in good health up to the present time. Present illness commenced with a severe cold. He has more or less dyspnœa, expectoration and pain in the chest.

On physical examination he was found to be well nourished; lips bluish; tongue heavily coated with white fur; pupils normal; subcrepitant and dry râles over entire chest; heart normal.

This was a case of lobar pneumonia, involving the lower lobes of both lungs. His temperature at the time of admission was 101°; respiration, 34; pulse, 120. At 12 o'clock the next afternoon, temperature was 103.4°; respiration, 35. Admitted into the hospital on the 5th of March, and two days after spat up several blood casts, some of them being very large, from the bronchial tubes. His temperature remained until the ninth day from 102.3° to 103°; most of the time being about 102°; respiration, 36 to 37.

The treatment he had received was the sulphate of strychnia, one-fortieth of a grain every four hours; also the cotton and oil silk jacket placed around the chest. On the 21st he was given fifteen grains of antipyrine when the temperature was 103.8°. In four hours after the administration of the antipyrine, temperature was 102.6°. At 4 p. m., on the 9th of the month, his temperature rose to 104°. He was then given another dose of fifteen grains of antipyrine, which promptly reduced the temperature 2°. On the 10th all the medicine stopped except the strychnia and whisky. We give whisky in nearly all cases of pneumonia. This patient had been taking a teaspoonful every two hours. On the 10th of March he developed great dyspnœa; breathing was rapid and shallow. It seemed almost impossible for him to get a sufficient amount of air. He was also hoarse, having some laryngeal trouble. Some blankets were now arranged around the bed so as to form a tent, the patient being allowed to remain in that tent with steam introduced into it, so that the air he breathed was moist and warm. The next day he was given fifteen grains tincture of iron, and one sixty-fourth of a grain of bichloride of mercury every four hours, as an alterative. Patient remained in the tent March 10th, 11th and 12th, until about 8 p. m., when the iron, bichloride of mercury and steam were all stopped, and patient felt a great deal better. On the evening of the 11th his temperature fell to 99.8°

If we date the sickness from the time until the temperature fell, we would say the crisis occurred on the ninth day, his respiration at this time being 24 to 27 and his pulse 94. He was given egg-nogs, good nourishment, and sulphite of calcium three times a day. His temperature remained the same up to the present time. He has no fever, and is now taking a mixture composed of cod-liver oil, syrup of hypophosphites and the iodide of iron.

PNEUMONIA—LEFT LOWER LOBE RECURRING IN RIGHT UPPER LOBE.

CASE 2.—This man was admitted to the hospital on the 24th day of January. Age 25; no history obtainable. Diagnosis, *pneumonia of the left lower lobe.* On admission, his pulse was 105; temperature, 102.2°; respiration, 24. Next day, pulse, 88; temperature, 102.4°; respiration, 40. Patient was placed on a light diet—egg-nogs, cotton and oil silk jacket placed around the chest, and given carbonate of ammonia and powdered camphor, each three grains, every two hours, with half an ounce of whisky. His temperature remained in the neighborhood of 101° and 102° until the 27th (being three days after admission), when it fell to 100°; respiration 24, gradually becoming less. On the 31st of January we heard a friction sound at the end of respiration below and inside of the left nipple. This localized pleurisy did not give rise to any particular trouble, and on the 14th of February he was discharged. On the 10th of March he was re-admitted with the following history: Family history, good; personal history, had pneumonia in January of this year; occupation necessitates much exposure. His present illness commenced with a severe chill last evening, followed by fever and profuse perspiration, diarrhœa commencing at the same time; stools watery; pain in the right side; severe frontal headache; skin soft and moist; pupils equal; tongue coated and flabby. Examination of the chest shows crepitant râles over right upper lobe of lung; heart normal; abdomen normal.

At the time of admission his pulse was 110; temperature, 102;° respirations, 28. Next evening, at 8 p. m., pulse 112; temperature, 103.4°; respirations, 24. This was a case of pneumonia of the right upper lobe, and his temperature ranged from 100 to 104° until the 16th of this month, when it became 99.6°, and it has not risen since.

On the 12th of the month a pleuritic friction sound was heard in the infra-mammary and axillary regions on the right side. Hot fomentations were applied, and ten grains of antipyrine given, to be repeated if needed. No further treatment was necessary. The treatment has been principally anodyne in its character to allay pain. At the present time he is getting whisky every four hours, and a light diet, with morphia when necessary.

This case is interesting on account of the fact that he has had two attacks of pneumonia within so recent a period, one involving the *lower left lobe* and the other the *upper right lobe.*

We now get a physical sign about an inch above the right nipple, known as the crepitant râle redux. A great many times the question is asked, how do you tell a crepitant râle from a subcrepitant? And the only distinction sometimes is the manner in which you hear the crepitant râle. In listening over the chest just above the nipple I hear a fine crackling sound at the close of inspiration. I do not hear it during expiration, nor at the beginning of inspiration. The sound we get in this case is similar to a friction sound—in fact so similar that it might be taken for one, and yet occurring at the end of inspiration, I have no hesitation in saying that it is a crepitant râle. It is an excellent example of the crepitant râles that we get in the first stage of pneumonia or the early stages of consumption.

### DUUBLE LOBULAR PNEUMONIA.

CASE 3.—This patient, a male, aged 40, worked in a lumber yard, native of Sweden, was admitted to the hospital on the 7th of March, No family history. Personal history good; no venereal disease. Present complaint commenced four days before admission with pain in the left side; patient expectorated freely; pupils equal; tongue heavily coated with fur in the center; crepitant and subcrepitant râles heard over both lower lobes; dullness on percussion; pulse on admission was 132; temperature 99.6°; respiration, 42.

TREATMENT.—Patient placed upon half an ounce of whisky every two hours, carbonate of ammonia, camphor three grains every six hours, milk diet, and later on one-fortieth of a grain of strychnia every four hours; cotton and oil-silk jacket applied. Same evening temperature was 102.6°; pulse, 122; respiration, 52, being quite shallow. After this date temperature ranged from 98.6° to 99.8° until midnight the next day, when it arose to 100.6°.

Temperature at no time has been over 102.6°; consequently it is a case in which the crisis undoubtedly occurred about the time he was admitted. He had probably had the greater part of the fever before his admission here.

The interesting feature connected with the case is, that on admission he was almost moribund; it was thought he would not recover, yet by means of hypodermic injections of strychnia every two hours, and whisky every hour, re-action was established. It is a good illustration of what can be accomplished in the way of hypodermic medication and unrelaxing exertions. In many instances we probably would regard such a case as hopeless, and would be tempted to relinquish our endeavors, and yet this case illustrates the fact that we do not know what the result will always be, and if we keep on with our efforts of treatment, especially by using strychnia and digitalis hypodermically, we can often tide our patients over the crisis. Probably bringing him to the hospital had something to do with the condition of collapse, and by resorting to the administration of whisky hypodermically he was tided over, so that he began to recover, and the result is, he is here to-day. Let me impress this maxim on your minds: Never cease your endeavors to keep the vital spark alive until you are absolutely sure it has been extinguished. I have seen so many apparently hopeless cases saved that I wish to impress this on your minds.

PNEUMONIA—TYPHOID SYMPTOMS.

CASE .4—John M., aged 29, ill three days before admitted to the hospital; admitted March 10th; mother died of consumption; smokes and drinks; works out doors; present illness commenced with pain in the left side of chest and back, followed by coughing and expectoration, sputum being at times streaked with blood; diarrhœa, with pain in the abdomen; frontal headache; skin moist; tongue moist and flabby; heart normal; abdomen tender on pressure, with gurgling in the right iliac fossa. On admission his pulse was 100; temperature 101°; respirations, 32. Cotton and oil-silk jacket was placed on chest. He was given carbonate of ammonia and powdered camphor, each 3 grains, with half an ounce of whisky every four hours. On the 12th whisky was stopped. Complication of typhoid fever was suspected in the case. The diagnosis, however, was *pneumonia of the left lower lobe*, as the typhoid symptoms gradually disappeared.

REMARKS.—There is one fact in connection with these cases that we must not overlook—we do not overtreat them. In nearly all of the cases we give whisky and strychnia, the one-fortieth of a grain of the latter every four hours. When the temperature rises over 103°, we give 15 to 20 grains of antipyrine. I believe every case that has entered the hospital since the 1st of January has had a cotton jacket placed on him, and there has only been one case which has been treated with poultices, and that only for a short time. The cotton jacket accomplishes the following objects: First, it prevents variations in the temperature of the chest; second, it is a source of great relief from pain by restraining the movements of the chest; and third, it favors perspiration. The patient who wears a cotton jacket will perspire freely, and we get the same action in relieving pulmonary hyperæmia we would get from poultices, with the advantage that we do not need to change it. Believing pneumonia to be a self-limited disease, the treatment consists principally in administering diffusible heart stimulants and easily digestible nourishment, the latter being chiefly milk.

The only source of danger in general in pneumonia is either from high temperature, or else failure of the heart. It has previously been the custom to give large doses of quinine to reduce the hyperexia, yet we give in this hospital antipyrine in preference to quinine, believing that it reduces the temperature more promptly, and not believing it has any injurious effect upon the heart when cautiously administered, although a great many physicians think it has. In case there are indications of heart failure, we give such cardiac stimulants as carbonate of ammonia, strychnia, digitalis or camphor.

I have jotted down some figures giving the percentage of cases of pneumonia that died in various hospitals, the list being taken from "Loomis' Practice." Out of 12,421 cases in the hospitals at Stockholm, 11 per centum died; Vienna, 24 per centum; Basle, 23 per centum; of the number of deaths in the late war of the United States army, white troops, 34 per centum; colored, 33 per centum; Bellevue Hospital statistics, extending over four years, of 255 cases, 34 per centum died.

In private practice we see that the percentage with different physicians varies somewhat. For instance, Lebert, out of 205 cases, lost 7.3 per centum; Ziemssen, 3.1 per centum; Bruhn, of Copenhagen, 20.1; Fox, 20; Walsh, 33.3; Bennett, out of 105 cases, lost none.

In this hospital, since January, we have had 41 cases of pneumonia with six deaths, being about 14.5 per centum. Three of the fatal cases died within twenty-four hours from the time of admission.

The character and seats of the inflammation in the 41 cases were as follows: Lobar pneumonia, involving one lower lobe, 31 cases; involving two lower lobes, 4 cases; involving upper right lobe, 1 case; involving both upper and lower right lobes, 1 case; involving right middle lobe, 1 case; lobular pneumonia, 3 cases. Typhoid fever was a complication in 3 cases and alcoholism in four cases. Gentlemen, you will readily see why we cannot get as low a percentage of mortality in pneumonia in hospital cases as in private cases, as the patients come from that class who not only transgress the moral and physical laws, but also hygienic laws. While we cannot hope to attain the marvelous success of Bennett in his 105 cases, we feel like congratulating ourselves that the mortality has been so low in our wards during the past three months.

